# Supplementary material for: ConVarT: a search engine for matching human genetic variants with variants from non-human species
Source: Nucleic Acids Res. 2021 Oct 28;50(D1):D1172–8. doi: 10.1093/nar/gkab939 (PMC8728286; doi:10.1093/nar/gkab939)

AP1M1-201, AP1M1 (GeneID: 8907) | Homo sapiens

Description:	adaptor related protein complex 1 subunit mu 1 [Source:HGNC Symbol;Acc:HGNC:13667]
Synonyms:	*AP1M1-201, MU-1A, AP47, CLTNM, CLAPM2*
Other ID(s):	HGNC:13667, ENSG00000072958
Protein Accession Numbers:	ENST00000291439, ENST00000444449,NP_115882,NP_001123996
Statistics:	COSMIC(162) dbSNP(14) PTM(32)

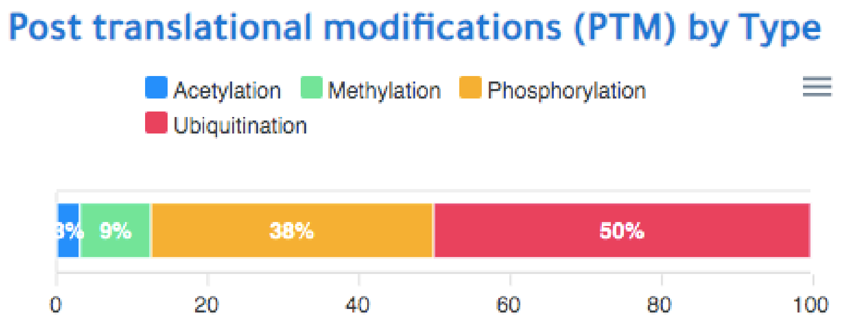

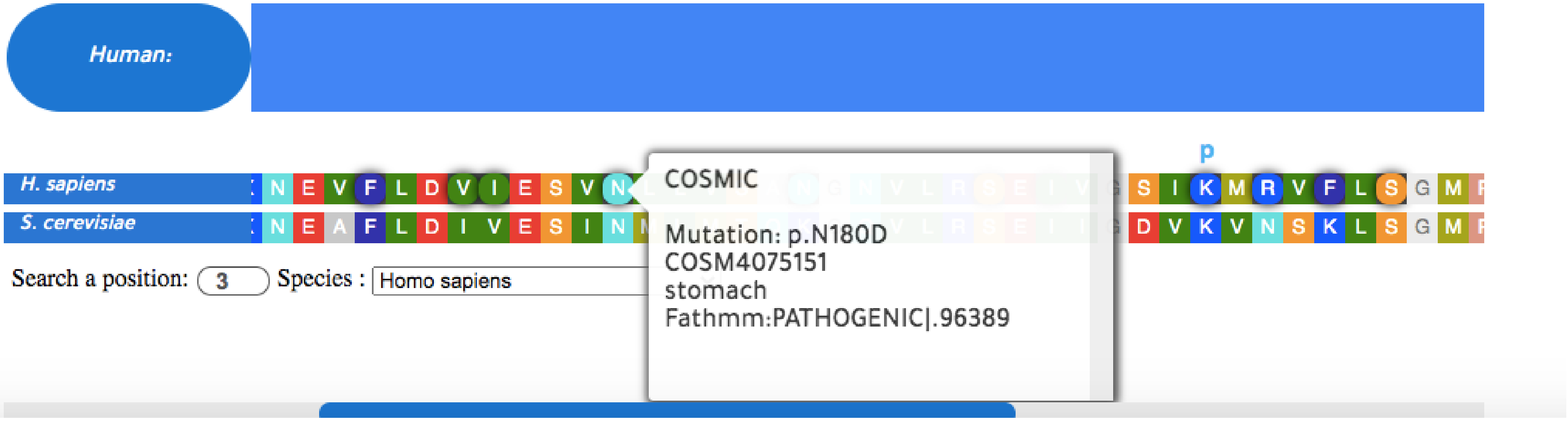

Supplement: gkab939_Supplemental_Files [file gkab939_supplemental_files.zip › Figure S2.pdf]
