# Supplementary figures and images for: ConVarT: a search engine for matching human genetic variants with variants from non-human species
Source: Nucleic Acids Res. 2021 Oct 28;50(D1):D1172–8. doi: 10.1093/nar/gkab939 (PMC8728286; doi:10.1093/nar/gkab939)

A

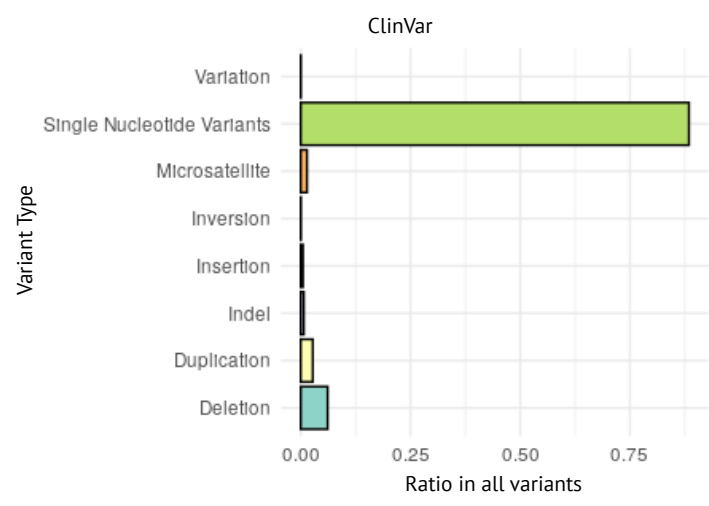

B

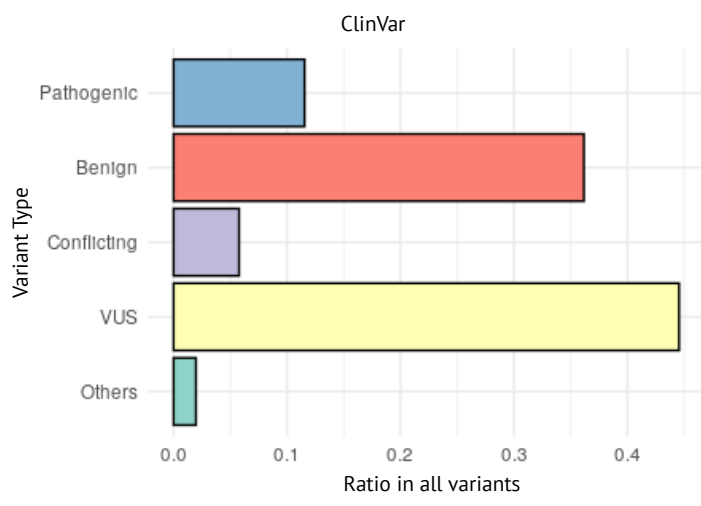

Supplement: gkab939_Supplemental_Files [file gkab939_supplemental_files.zip › Figure S1.pdf]
